# Supplementary material for: The defensome of complex bacterial communities
Source: Nat Commun. 2024 Mar 8;15:2146. doi: 10.1038/s41467-024-46489-0 (PMC10924106; doi:10.1038/s41467-024-46489-0)
Supplement: Supplementary file 1 — Supplementary Information [file 41467_2024_46489_MOESM1_ESM.pdf]

The defensome of complex bacterial communities
(Supplementary Information)

Angelina Beavogui[1], Auriane Lacroix[1], Nicolas Wiart[2], Julie Poulain[1,3], Tom O. Delmont[1,3], Lucas Paoli[4,5], Patrick Wincker[1,3], Pedro H. Oliveira[1, #]

[1]Génomique Métabolique, Genoscope, Institut François Jacob, Commissariat à l'Energie Atomique (CEA), CNRS, Université Evry, Université Paris-Saclay, 2 Rue Gaston Crémieux, 91057 Evry, France.

[2]Genoscope, Institut François Jacob, CEA, Université Paris-Saclay, 2 Rue Gaston Crémieux, 91057 Evry, France.

[3]Research Federation for the Study of Global Ocean Systems Ecology and Evolution, FR2022 / Tara GOsee, Paris, France.

[4]Department of Biology, Institute of Microbiology and Swiss Institute of Bioinformatics, ETH Zürich, Zürich 8093, Switzerland

[5]Institut Pasteur, Université Paris Cité, INSERM U1284, Molecular Diversity of Microbes lab, Paris, France

[#] To whom correspondence should be addressed. Pedro H. Oliveira (pcoutool@genoscope.cns.fr)

Supplementary Figures

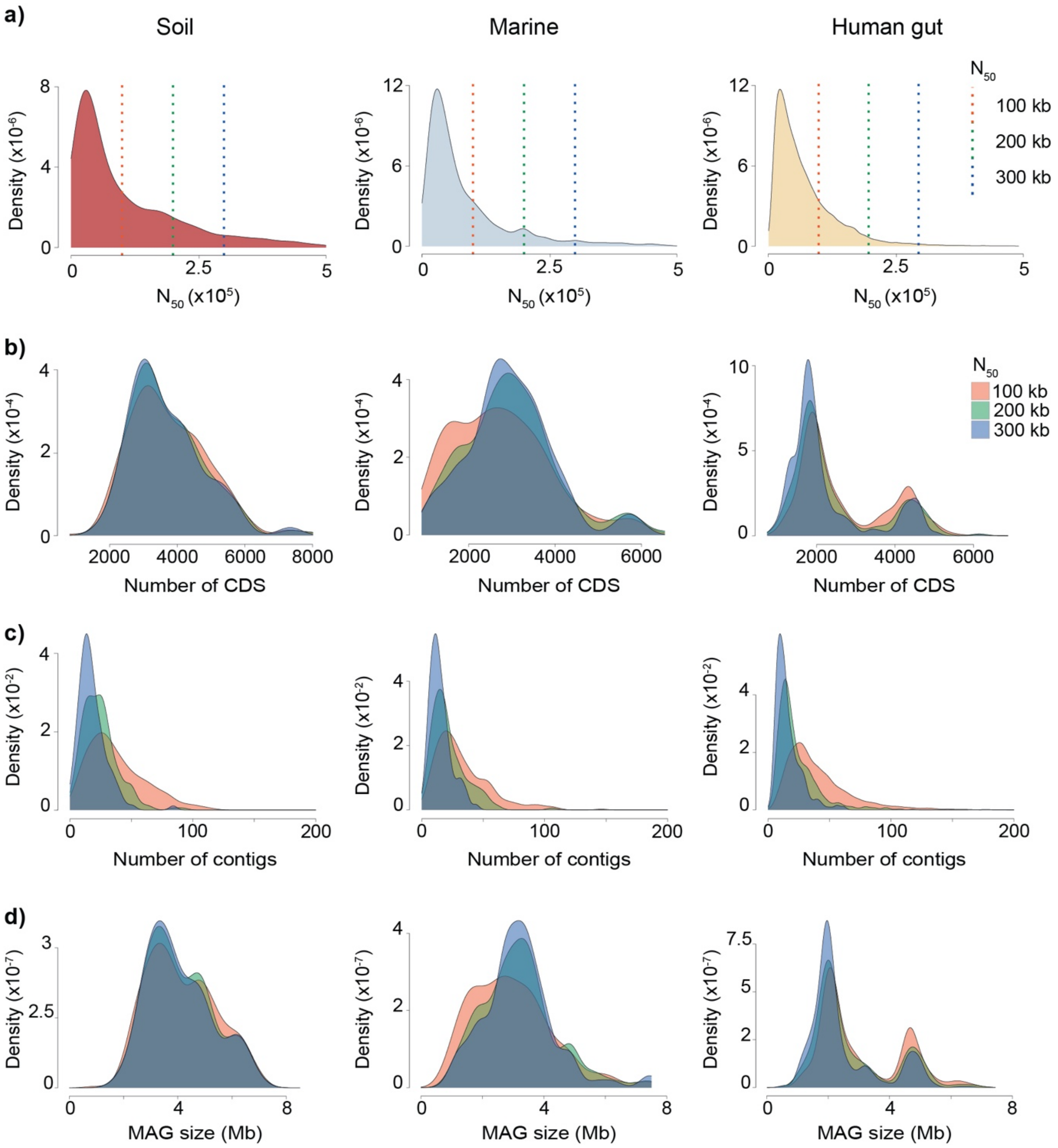

Supplementary Fig. 1. Density distributions of key MAG parameters according to environment: (*a*) N_{50}; (*b*) number of CDS; (*c*) number of contigs; and (*d*) size. Source data are provided as a Source Data file.

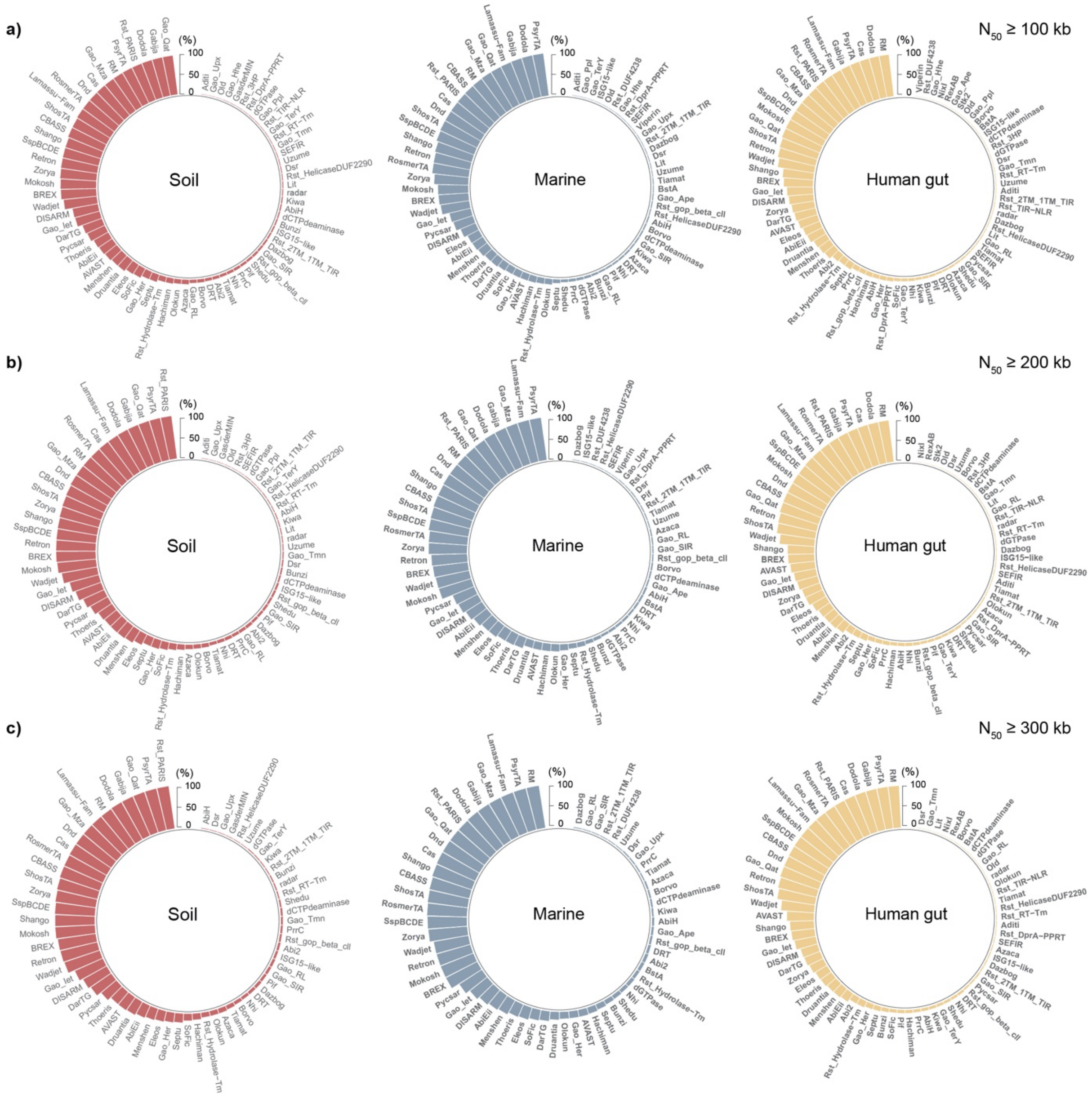

Supplementary Fig. 2. Percentage of soil, marine and human gut MAGs harboring each family of defense genes. Shown are assemblies with values of N_{50} ≥ 100, 200, and 300 kb. Source data are provided as a Source Data file.

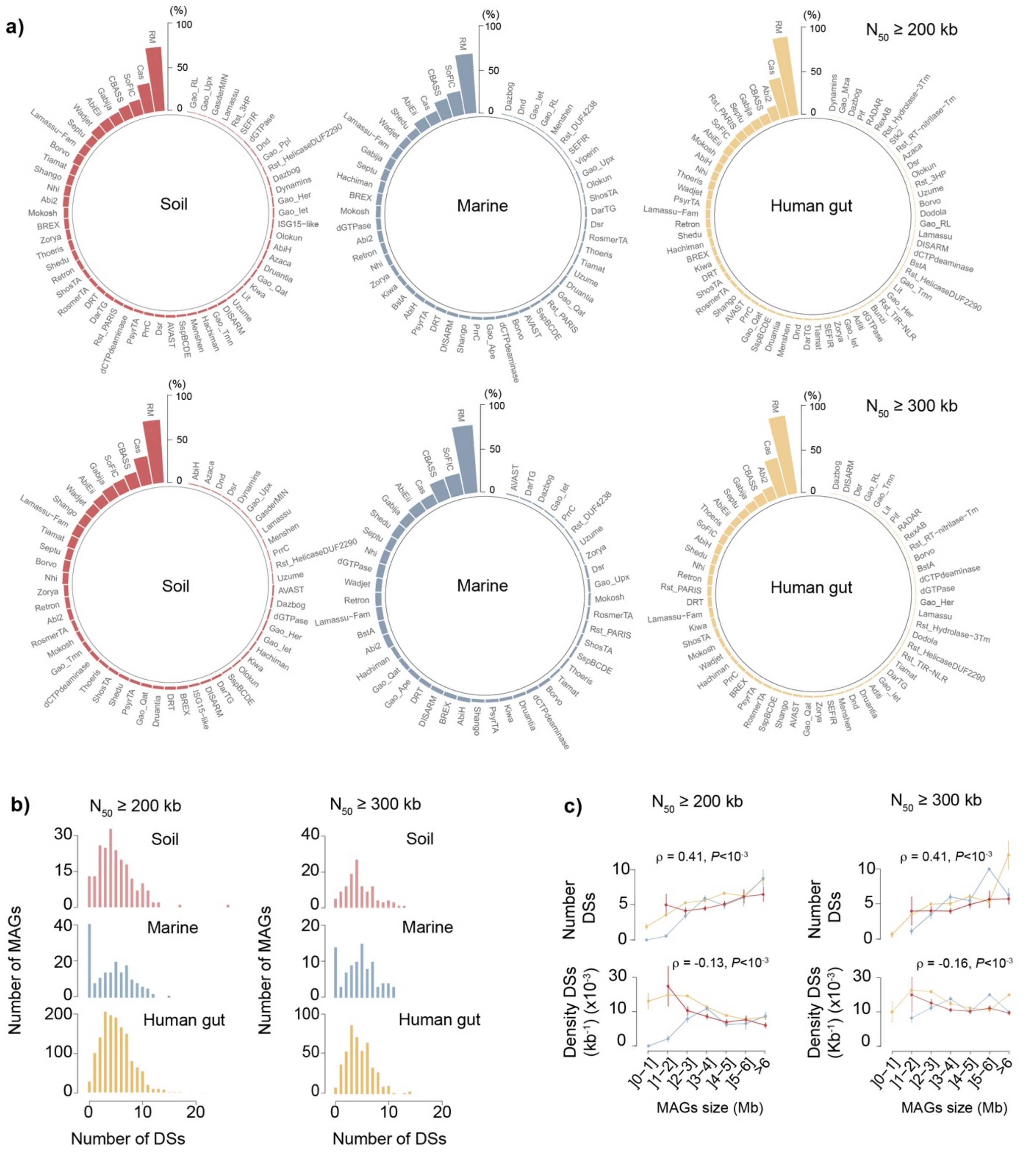

Supplementary Fig. 3. Distribution of defense systems in MAGs. (*a*) Percentage of soil, marine and human gut MAGs harboring each family of defense system. Shown are assemblies with values of N_{50} ≥ 200 and 300 kb. (*b*) Distribution of number of defense systems (DSs, per MAG) across environments for assemblies with values of N_{50} ≥ 200 and 300 kb. (*c*) Variation of number and density (per kb) of defense systems (DSs) with MAG size (Mb) for assemblies with values of N_{50} ≥ 200 and 300 kb and for each environment (*n* = 214 and *n* = 121; *n* = 172 and *n* = 90; *n* = 1,429 and *n* = 457 MAGs from soil, marine, and human gut and N_{50} ≥ 200 and 300 kb respectively). Error bars represent standard deviations of the mean and correlation was evaluated by a two-sided Spearman's rank test. Source data are provided as a Source Data file.

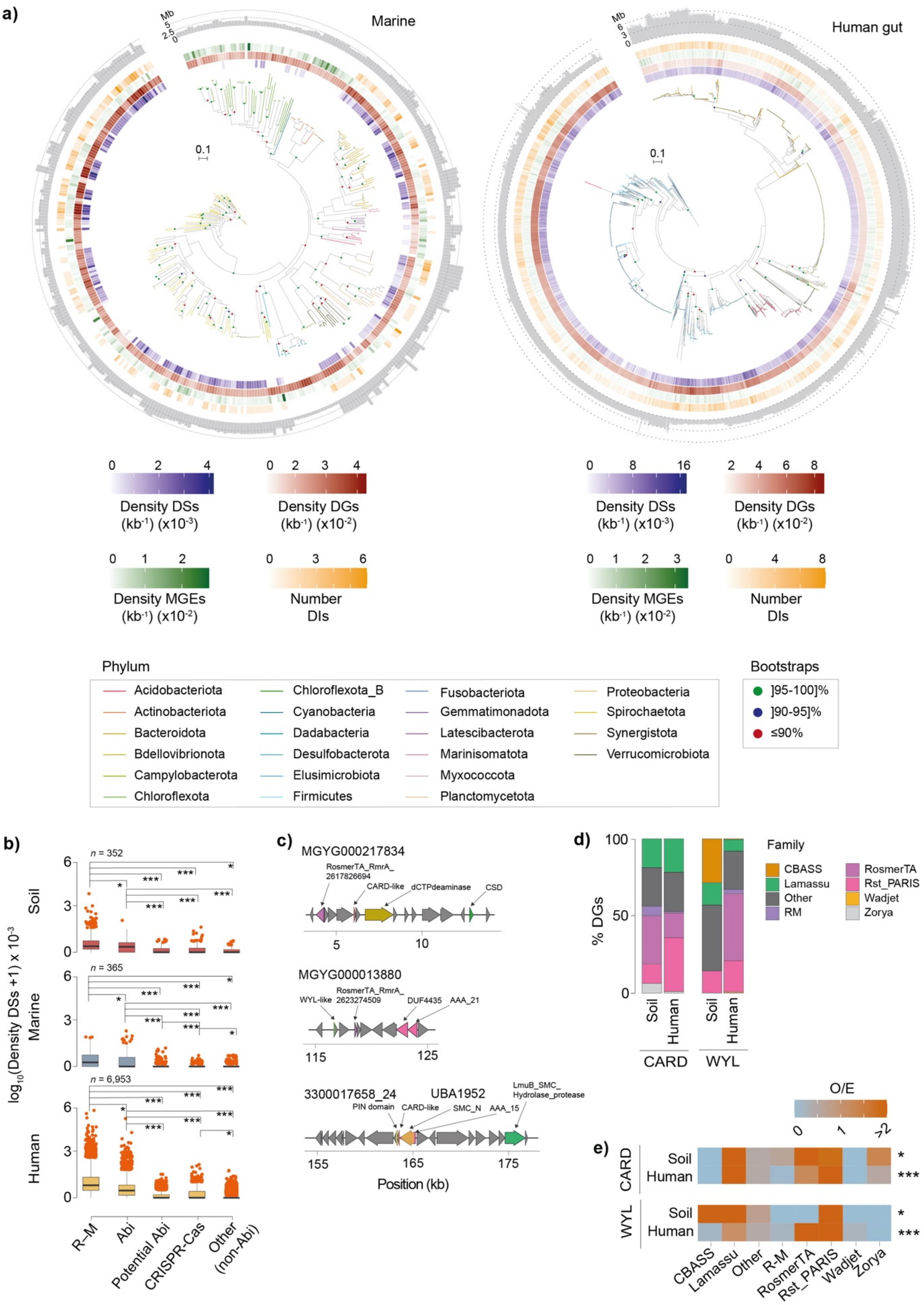

a)
Marine
Human gut
Mb
0.1
Density DSs (kb-1) (x10-3)
Density DGs (kb-1) (x10-2)
Density MGEs (kb-1) (x10-2)
Number DIs
Phylum
Acidobacteriota
Actinobacteriota
Bacteroidota
Bdellovibrionota
Campylobacterota
Chloroflexota
Chloroflexota_B
Cyanobacteria
Dadabacteria
Desulfobacterota
Elusimicrobiota
Firmicutes
Fusobacteriota
Gemmatimonadota
Latescibacterota
Marinisomatota
Myxococcota
Planctomycetota
Proteobacteria
Spirochaetota
Synergistota
Verrucomicrobiota
Bootstraps
]95-100]%
]90-95]%
≤90%
b)
log10(Density DSs +1) x 10-3
Soil
Marine
Human
n = 352
n = 365
n = 6,953
R-M
Abi
Potential Abi
CRISPR-Cas
Other (non-Abi)
c)
MGYG000217834
RosmerTA_RmrA_ 2617826694
CARD-like
dCTPdeaminase
CSD
MGYG000013880
WYL-like
RosmerTA_RmrA_ 2623274509
DUF4435
AAA_21
3300017658_24
UBA1952
PIN domain
CARD-like
SMC_N
AAA_15
LmuB_SMC_ Hydrolase_protease
Position (kb)
d)
% DGs
Family
CBASS
Lamassu
Other
RM
RosmerTA
Rst_PARIS
Wadjet
Zorya
Soil
Human
CARD
WYL
e)
O/E
CBASS
Lamassu
Other
R-M
RosmerTA
Rst_PARIS
Wadjet
Zorya

Supplementary Fig. 4. Phylogenetic distribution of the defensome and association with types of defense mechanism and CARD / WYL domains. (*a*) Phylogenetic representation of 386 marine and 6,369 human gut MAGs, their corresponding phyla, density (per kb) of defense systems (DSs, purple), defense genes (DGs, red), MGEs (green), and number of defense islands (DIs, yellow). Distribution of MAG sizes (Mb) are shown as outer layer barplots. (*b*) Defense system (DS) density (per MAG per kb) split per biome and underlying defense mechanism (R–M, Abi, potential Abi, CRISPR-Cas, and other (non-Abi)). Boxplots represent the 25th to 75th percentiles, the inner black line marks the median, whiskers extend to 1.5x the interquartile range, and data points are set as outliers. Significance was tested by a two-sided Mann–Whitney–Wilcoxon test. Number of MAGs analyzed are shown as *n*. (*c*) Representative instances of colocalized (less than 10 genes apart) WYL and CARD domain-containing genes with defense genes in the human MAGs MGYG000217834 and MGYG000013880. Also shown is a chromosomal region of the Bacteroidetes bacterium UBA1952 sharing similarity with a recently described *Pedobacter rhizosphaerae* CARD-encoding defense system. (*d*) Stacked barplots showing the relative abundance of families of defense genes that colocalize with WYL- and CARD-like domains. (*e*) Heatmap of observed / expected (O / E) ratios of colocalization between genes belonging to distinct defense families and WYL and CARD-like domains. Expected values were obtained by multiplying the total number of genes pertaining to a given defense family by the fraction of defense genes of that family colocalized with a given domain. Significance was tested by a two-sided Chi-square test. To avoid performing analyses with weak statistical power, we omitted the marine MAG dataset due to their low WYL and CARD domain abundance (detected in as much 0.75% of the dataset). *P* values are indicated as $*P < 0.05$; $***P < 10^{-3}$. Source data are provided as a Source Data file.

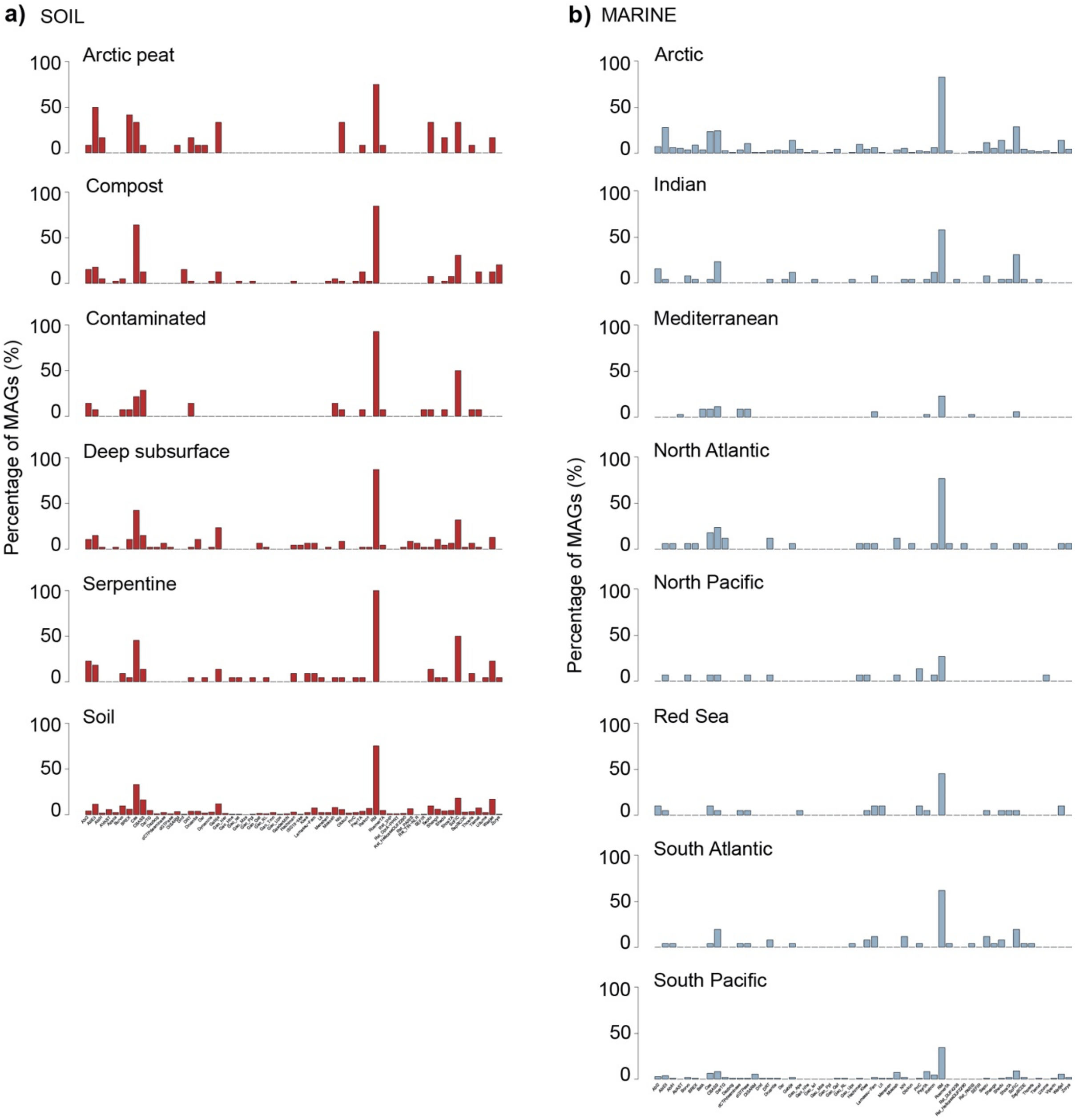
a) SOIL
Percentage of MAGs (%)
Arctic peat
Compost
Contaminated
Deep subsurface
Serpentine
Soil
100
50
0
b) MARINE
Percentage of MAGs (%)
Arctic
Indian
Mediterranean
North Atlantic
North Pacific
Red Sea
South Atlantic
South Pacific

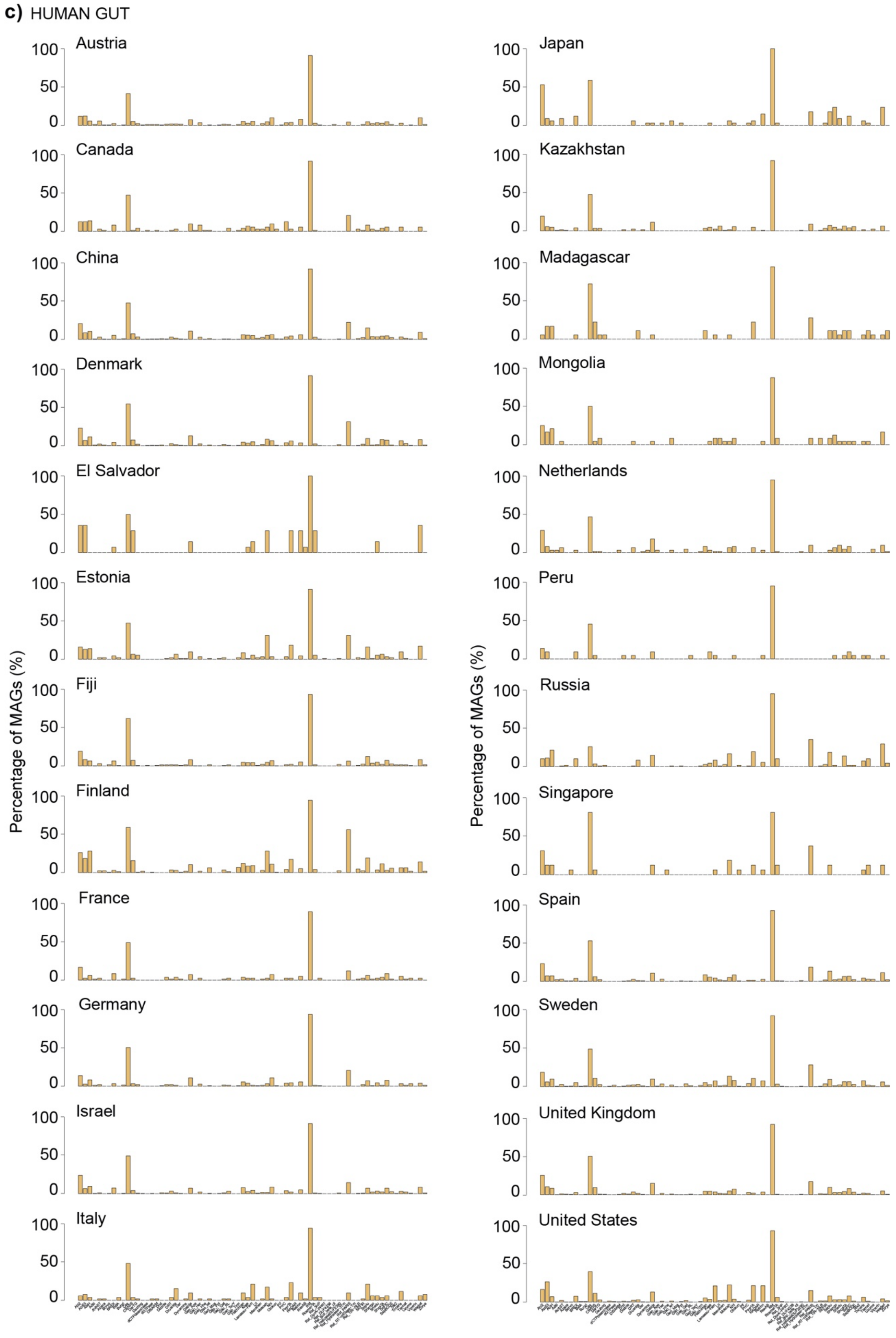

Supplementary Fig. 5. Percentage of (*a*) soil, (*b*) marine and (*c*) human gut MAGs harboring each family of defense system across different ecological and geographical backgrounds. Source data are provided as a Source Data file.

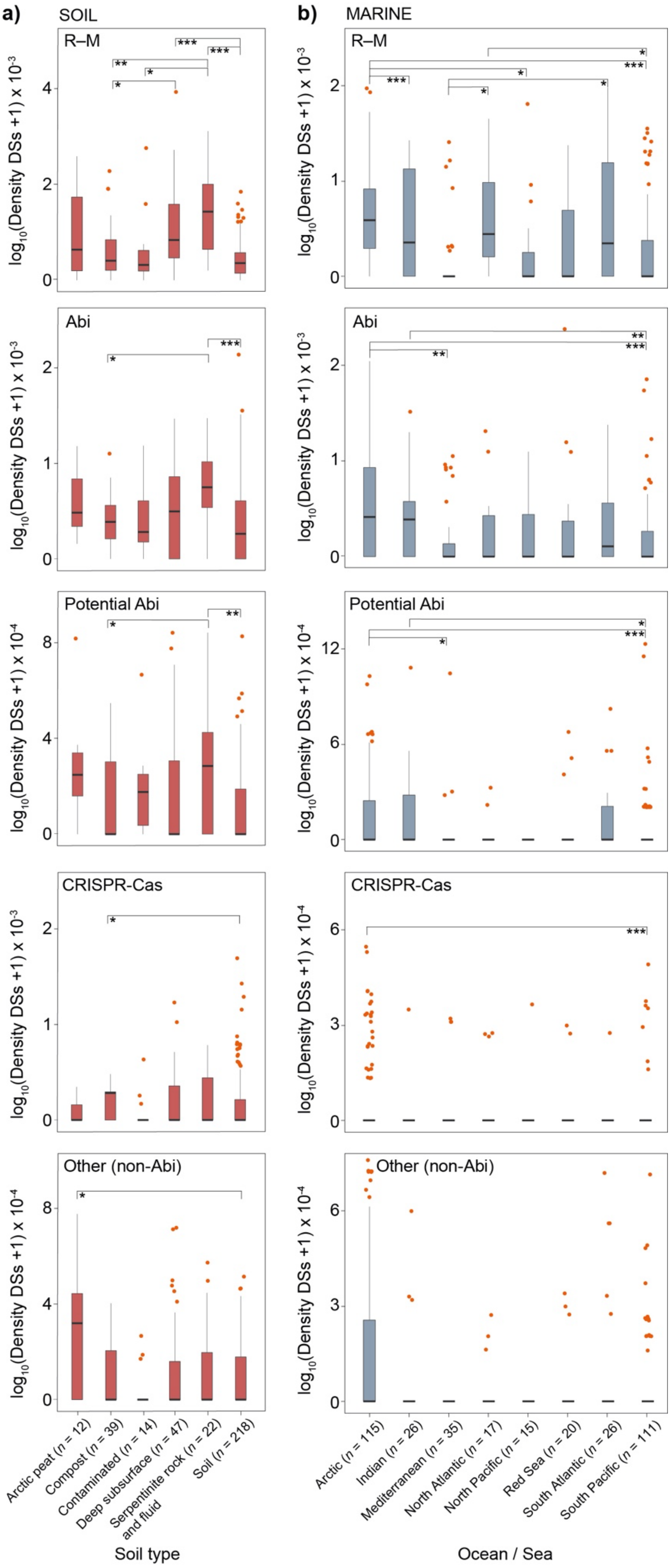

a)
SOIL
b)
MARINE
R–M
Abi
Potential Abi
CRISPR-Cas
Other (non-Abi)
log10(Density DSs +1) x 10-3
log10(Density DSs +1) x 10-4
Arctic peat (n = 12)
Compost (n = 39)
Contaminated (n = 14)
Deep subsurface (n = 47)
Serpentinite rock (n = 22) and fluid
Soil (n = 218)
Soil type
Arctic (n = 115)
Indian (n = 26)
Mediterranean (n = 35)
North Atlantic (n = 17)
North Pacific (n = 15)
Red Sea (n = 20)
South Atlantic (n = 26)
South Pacific (n = 111)
Ocean / Sea

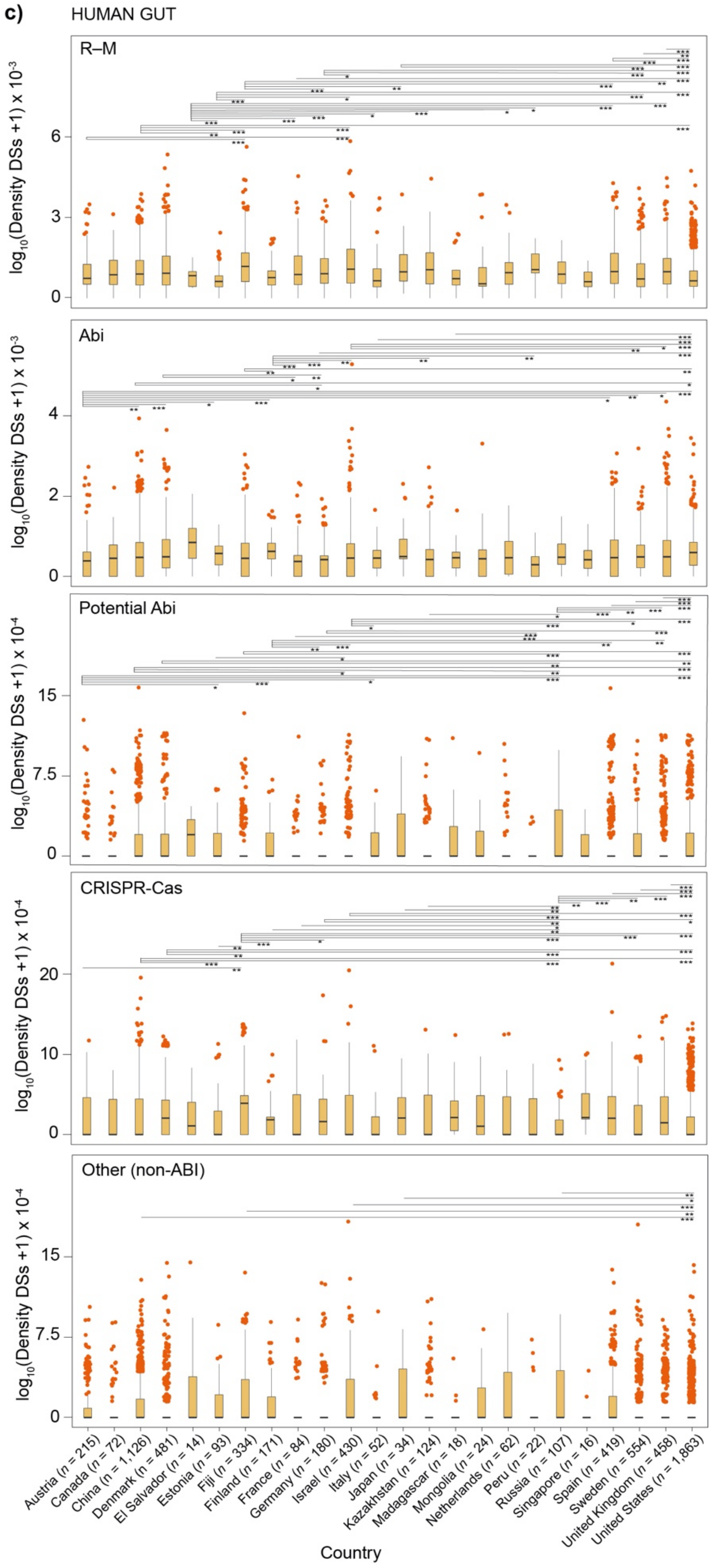

Supplementary Fig. 6. Defense system (DS) density (per MAG per kb) split according to defense mechanism (R–M, Abi, potential Abi, CRISPR-Cas, and other (non-Abi)), and different ecological (soil, marine) (*a*, *b*) and geographical (human gut) (*c*) contexts. Boxplots represent the 25th to 75th percentiles, the inner black line marks

the median, whiskers extend to 1.5x the interquartile range, and data points are set as outliers. Significance was tested by a two-sided Mann–Whitney–Wilcoxon test and *P* values are indicated as $*P < 0.05$; $**P < 10^{-2}$; $***P < 10^{-3}$. Number of MAGs analyzed are shown in parentheses. Source data are provided as a Source Data file.

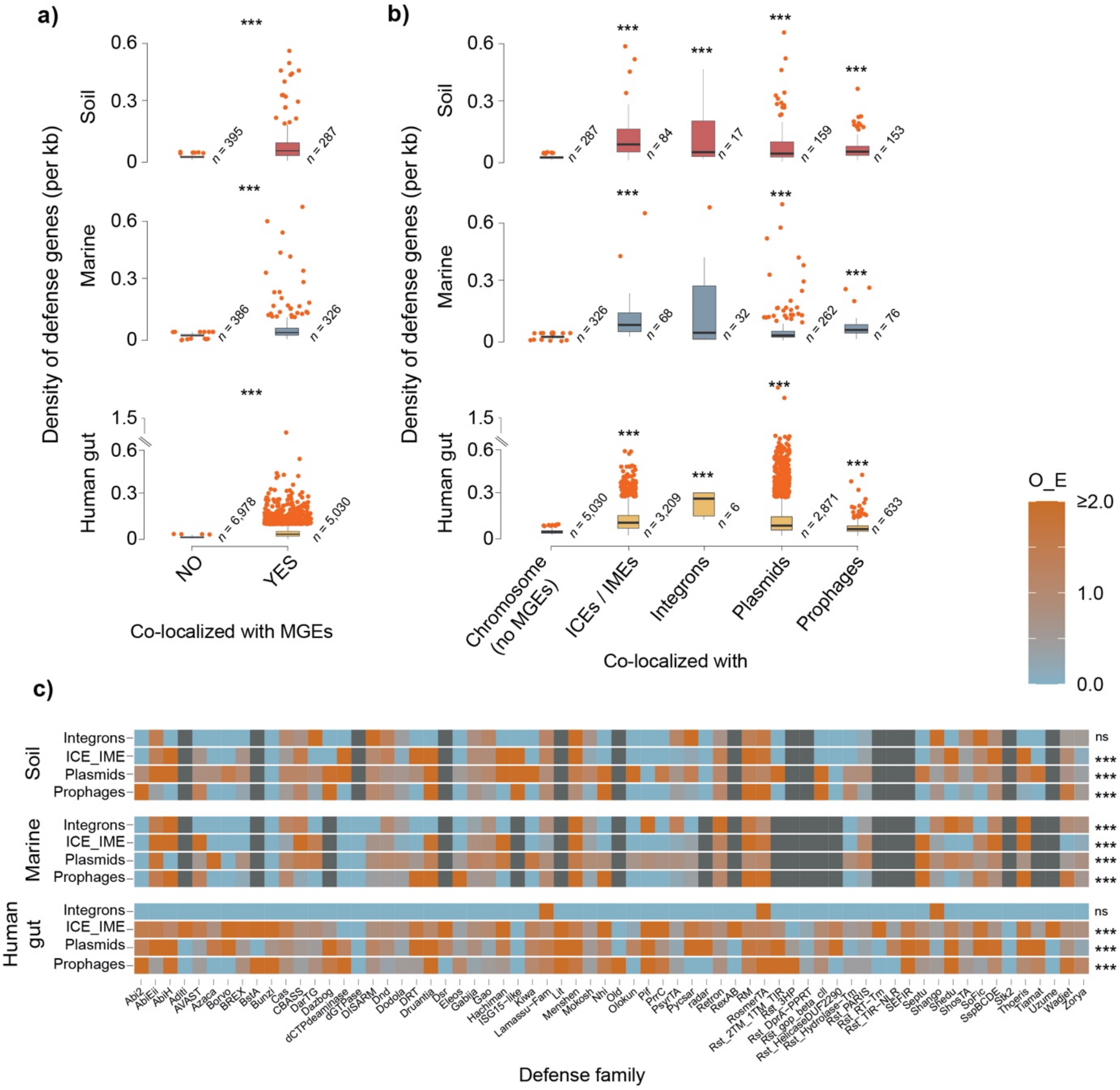

Supplementary Fig. 7. The genetic mobility of the defensome. Reproduction of **Fig. 4** of the manuscript with the inclusion of integrons. (*a*) Genomic colocalization of defense genes and MGEs. Number of MAGs analyzed are shown as *n* values. (*b*) Genomic colocalization of defense genes with plasmids, prophages, integrons, and ICEs / IMEs. (*c*) Heatmap of observed / expected (O / E) ratios of colocalization between genes belonging to distinct defense families and MGEs. Expected values were obtained by multiplying the total number of genes pertaining to a given defense family by the fraction of defense genes of that family assigned to each MGE. Dark gray squares represent absence of colocalization. Significance was tested by a two-sided Chi-square test. Boxplots represent the 25th to 75th percentiles, the inner black line marks the median, whiskers extend to 1.5x the interquartile range, and data points are set as outliers. Significance was tested by a two-sided Mann–Whitney–Wilcoxon test. *P* values are indicated as ***$P < 10^{-3}$. Source data are provided as a Source Data file.

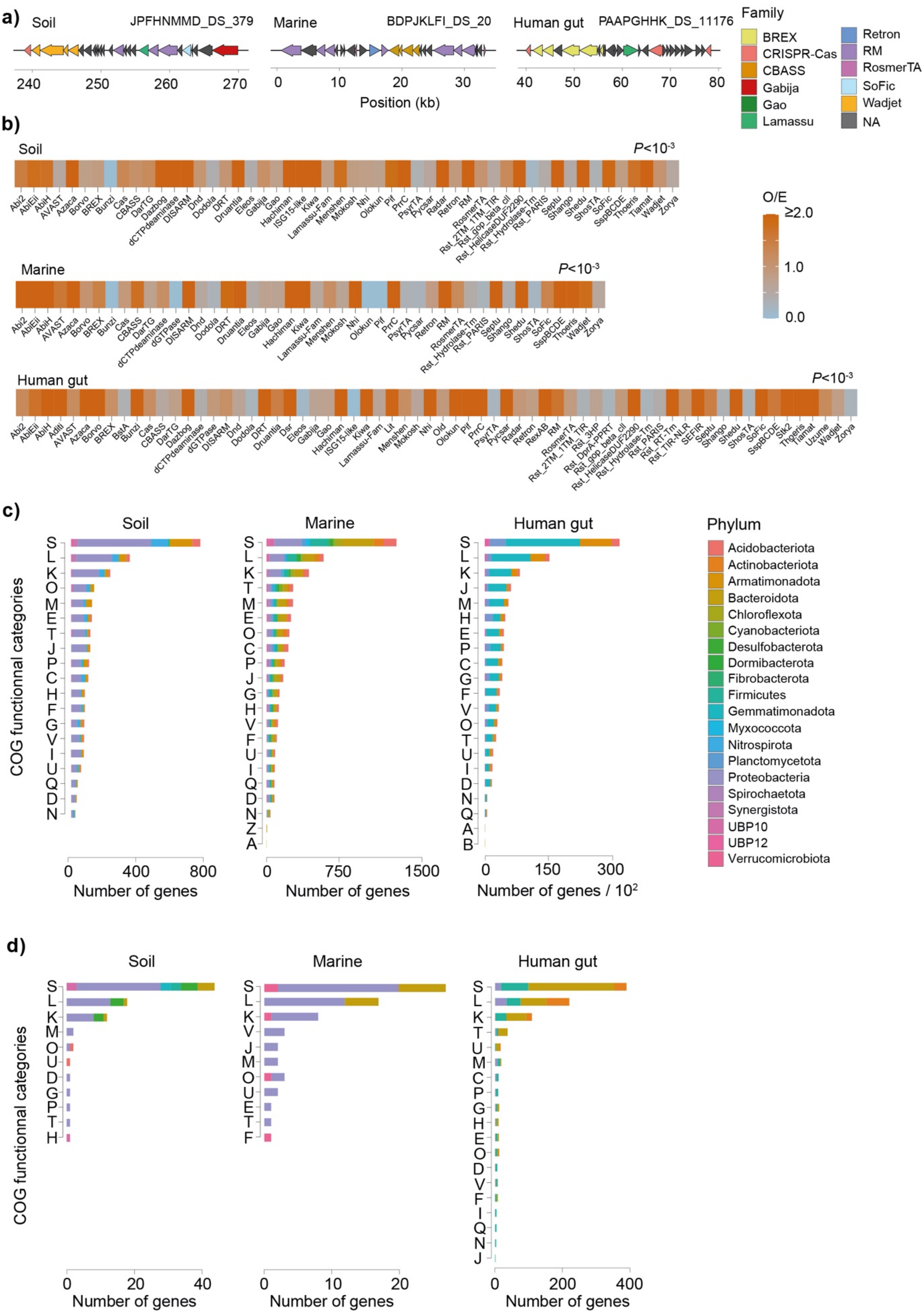

Supplementary Fig. 8. The MAG defense island repertoire. (*a*) Representative examples of defense islands for each environment, illustrating the high diversity of defense families present. (*b*) Heatmap of observed/expected (O / E) ratios of colocalization between genes belonging to distinct defense families and defense islands. Expected

values were obtained by multiplying the total number of genes pertaining to a given defense family by the fraction of defense genes of that family assigned to a defense island. Significance was tested by a two-sided Chi-square test. (*c*) COG functional annotation assessed by Eggnog-mapper of the ensemble of non-defensive genes in defense islands. (*d*) COG functional annotation of the ensemble of non-defensive genes in defense islands when complete defense systems were used as counting units for the classification of defense islands (see Methods). Source data are provided as a Source Data file.

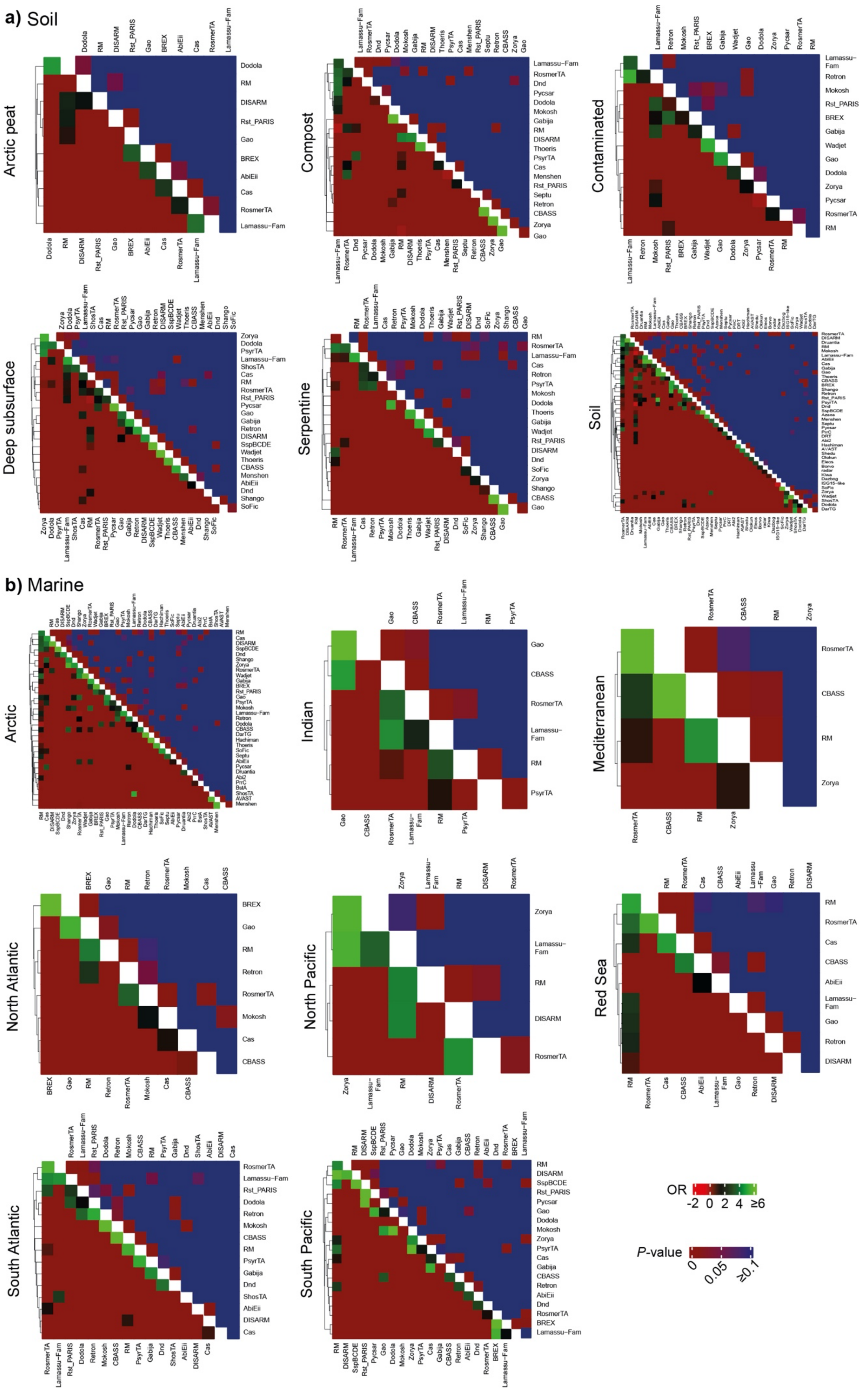
a) Soil
Arctic peat
Compost
Contaminated
Deep subsurface
Serpentine
Soil
b) Marine
Arctic
Indian
Mediterranean
North Atlantic
North Pacific
Red Sea
South Atlantic
South Pacific
OR
-2 0 2 4 ≥6
P-value
0 0.05 ≥0.1

c) Human gut

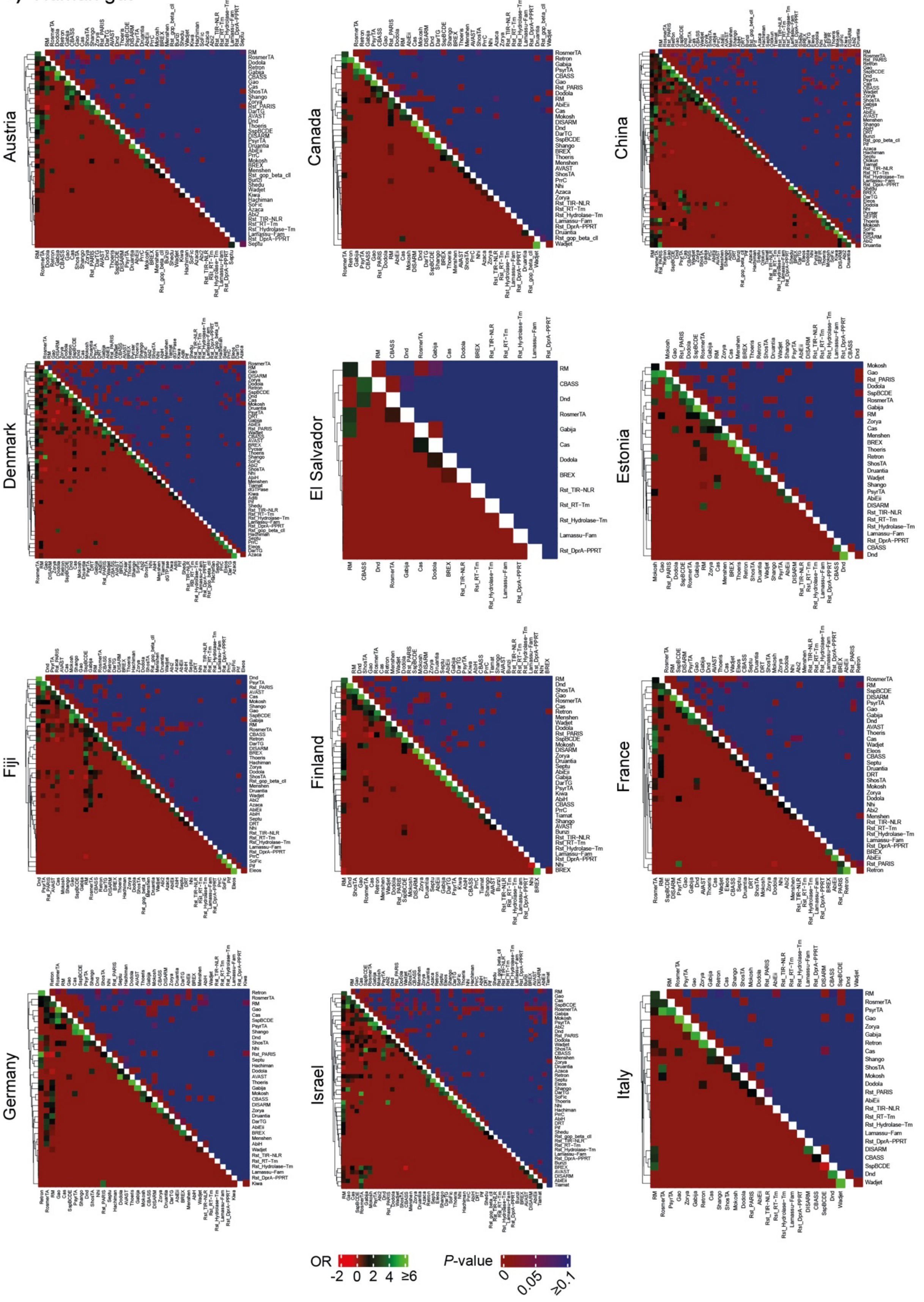
Austria
Canada
China
Denmark
El Salvador
Estonia
Fiji
Finland
France
Germany
Israel
Italy
OR
-2 0 2 4 ≥6
P-value
0
0.05
≥0.1

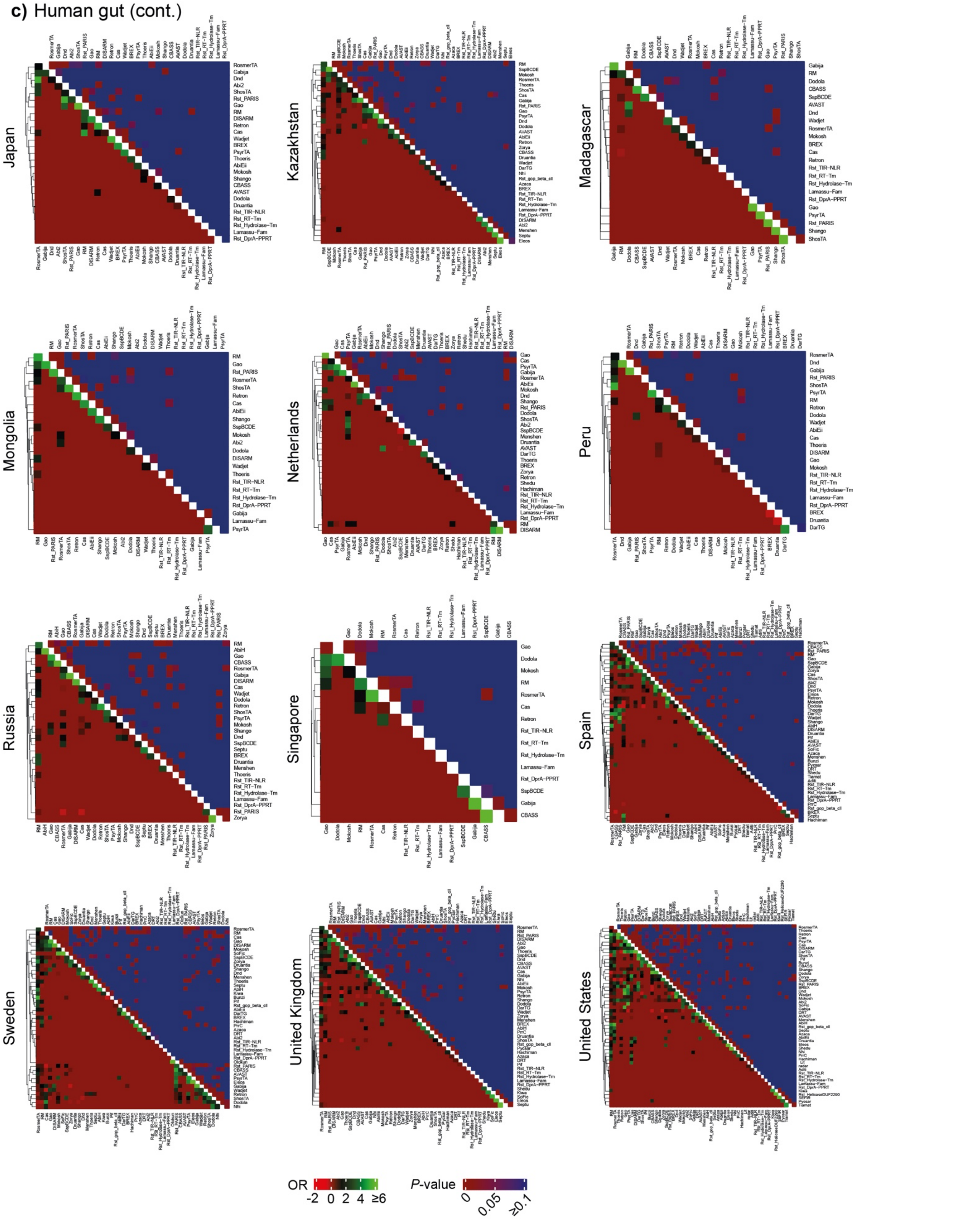

Supplementary Fig. 9. Defense families' odds ratio (OR) of colocalization in defense islands (bottom heatmaps) and associated two-sided Fisher's exact test *P* value (upper heatmaps) for different ecological (soil, marine) (*a*, *b*) and geographical (human gut) (*c*) contexts. Source data are provided as a Source Data file.

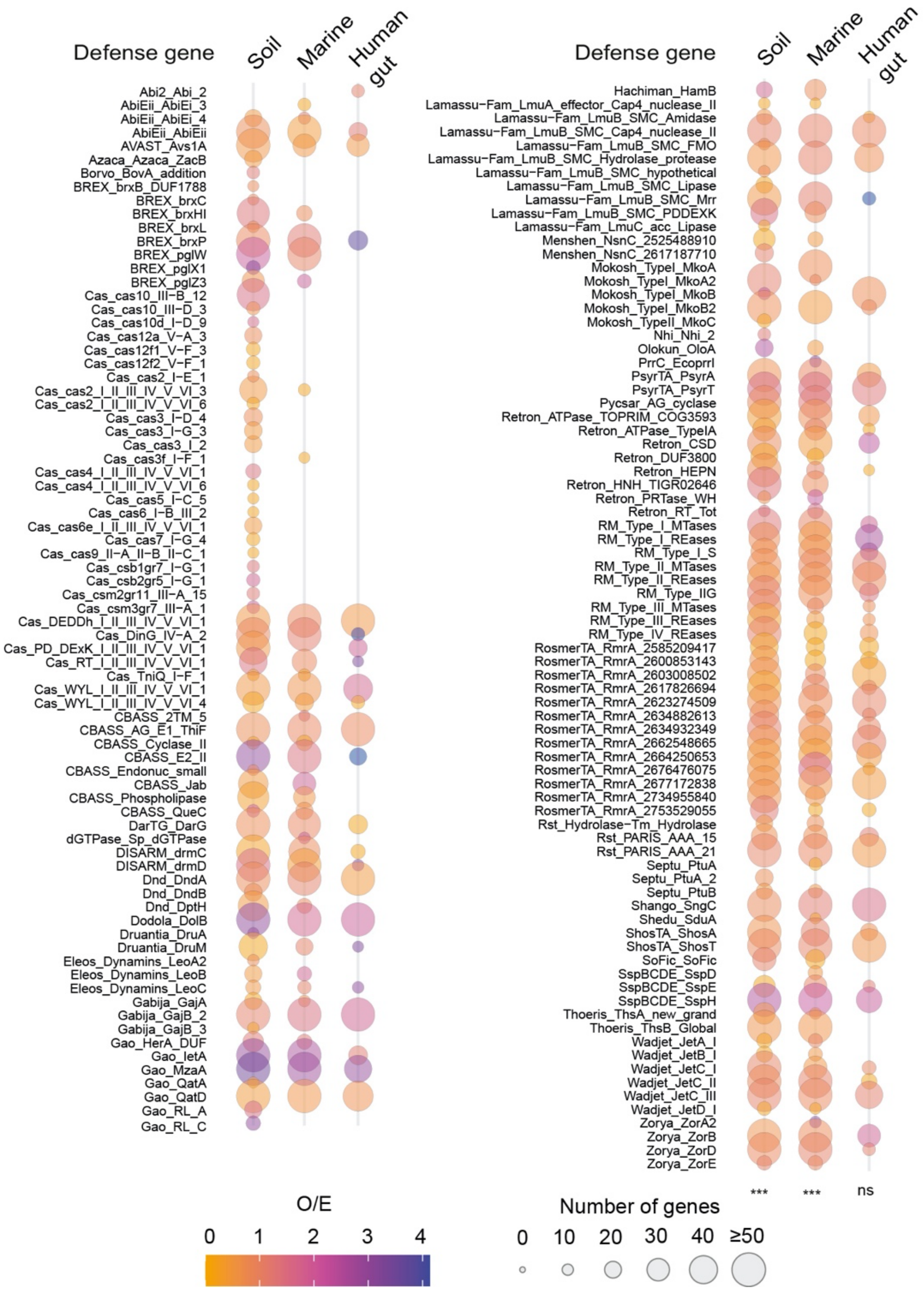

Supplementary Fig. 10. The genetic variability of the defensome. (*a*) 90 metagenomes (30 for each environment) having a broad representativity in terms of sampling sites (soil and marine) and countries (human gut), as well as in terms of presence of most defense families previously identified by DefenseFinder were selected. Shown in circles are the observed / expected (O / E) ratios of number of defense genes harboring high-frequency (≥ 25% of coverage at the variant position) SNPs + indels positions in their gene body (including 200 bp upstream the start codon). No thresholds on O / E ratio were introduced. Expected values were obtained by multiplying the total number of genes pertaining to a given defense family by the fraction of defense genes of that family harboring high-frequency alleles. Circle radius corresponds to the total number of defense genes analyzed per family. All defense families are represented irrespectively of their O / E ratio span. Significance was tested by a two-sided Chi-square test. *P* values are indicated as ***$P < 10^{-3}$; ns: not significant. Source data are provided as a Source Data file.

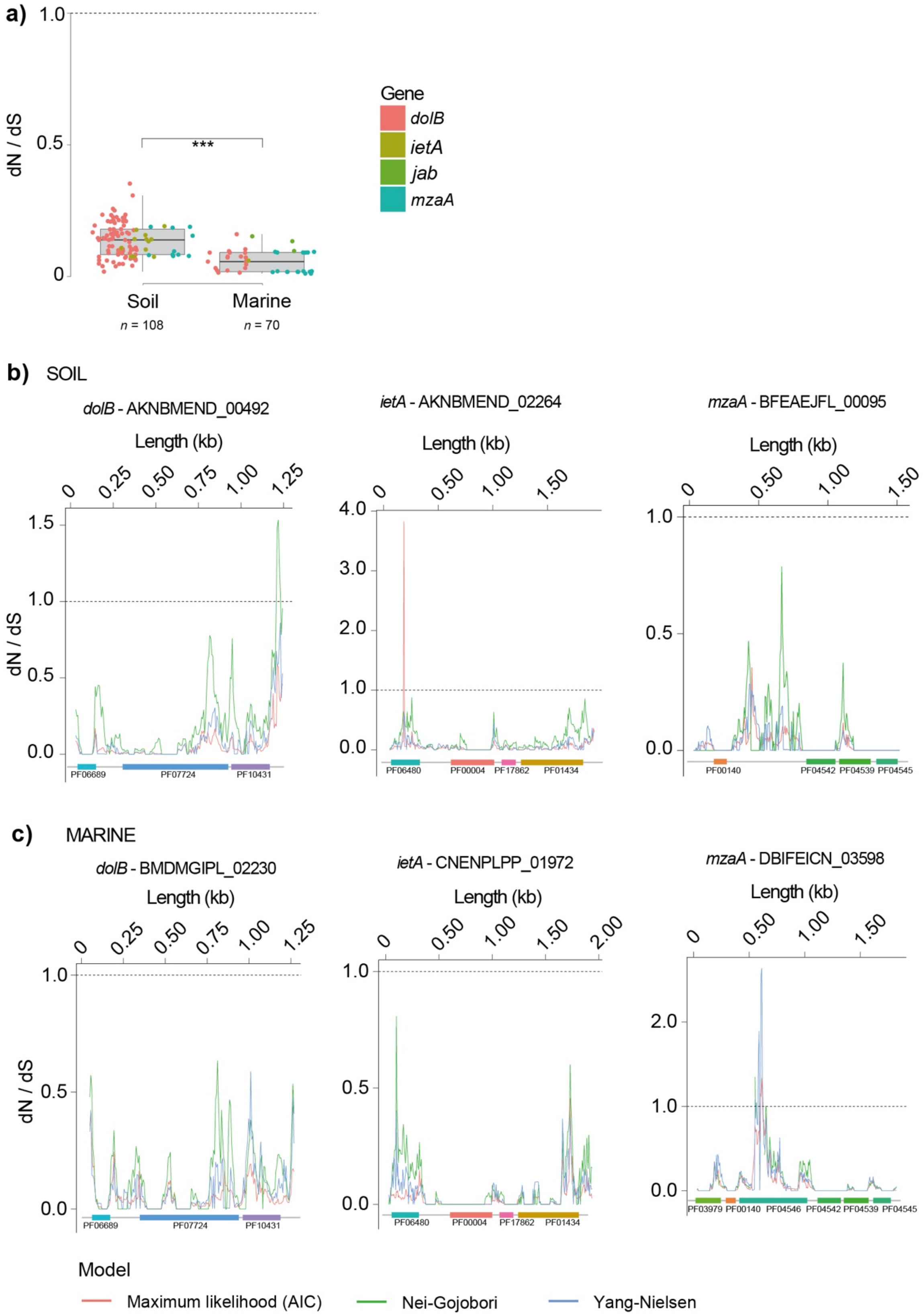

Supplementary Fig. 11. Evolution of defense genes. (*a*) Variation in global dN/dS given by the Nei-Gojobori (NG) and Yang-Nielson (NY) methods for a selection of defense genes shown to harbor a significantly higher frequency of SNPs + Indels. Boxplots represent the 25th to 75th percentiles, the inner black line marks the median, and whiskers extend to 1.5x the interquartile range. Number of genes analyzed are shown as *n* values. Significance

was tested by a two-sided Mann–Whitney–Wilcoxon test and *P* values are indicated as $***P < 10^{-3}$. (*b*, *c*) Across gene profiles of dN/dS given by the Maximum Likelihood (Akaike Information Criterium), Nei-Gojobori (NG), and Yang-Nielson (NY) methods for a selection of three defense genes simultaneously present in soil and marine environments. PFAM domains are shown as colored rectangles. Source data are provided as a Source Data file.
