# Supplementary material for: The defensome of complex bacterial communities
Source: Nat Commun. 2024 Mar 8;15:2146. doi: 10.1038/s41467-024-46489-0 (PMC10924106; doi:10.1038/s41467-024-46489-0)
Supplement: Supplementary file 3 — Description of Additional Supplementary Files [file 41467_2024_46489_MOESM3_ESM.pdf]

Description of Supplementary Data Files

Supplementary Data 1.
Description: Number of MAGs and unique taxa for different cutoffs of N50 ranging from 100-300 kb.

Supplementary Data 2.
Description: Most abundant taxa at the Phylum and Class levels for different cutoffs of N50 ranging from 100-300 kb. The top three taxa are colored in yellow.

Supplementary Data 3.
Description: Assembly statistics and metadata of the 7,759 high-quality non-redundant soil, marine, and human gut MAGs. All MAGs were filtered based on the Minimum Information about a Metagenome Assembled Genome (MIMAG) standard (≥100K N50, ≥90% completeness, ≤5% contamination, ≥18/20 tRNA genes and presence of at least one class of 5S, 16S and 23S rRNA genes). Shown is also information regarding taxonomy, habitat, number, and density (per kb) of defense genes (DGs) and defense systems (DSs).

Supplementary Data 4.
Description: Defense gene prediction as given by DefenseFinder. When multiple defense genes were predicted for the same CDS, only the best ranked hit (on the basis of E-value and hit coverage) was selected.

Supplementary Data 5.
Description: Maximum-likelihood phylogenetic trees (in Newick format) built from a concatenate of L2, L3, L4, L5, L6, L14, L16, L18, L22, L24, S3, S8, S10, S17, S19 ribosomal proteins.

Supplementary Data 6.
Description: Classification of defense families according to their defense mode (R-M, Abi, possible Abi, CRISPR-Cas, other non-Abi, and unknown).

Supplementary Data 7.
Description: Results of the stepwise linear regression analyses performed across the three environments using as response variable the number of defense genes (DGs) as a function of the predictor variables MAG size and phylogenetic depth (PD). SS: sum of squares; RSS: residual sum of squares; AIC: Akaike Information Criterium. Forward, backward, and forward-backward regression strategies were performed.

Supplementary Data 8.
Description: Percentage of MAGs containing each defense family across multiple habitats / geographical locations.

Supplementary Data 9.
Description: Percentage of MAGs containing each defense family across multiple habitats / geographical locations.

Supplementary Data 10.
Description: Genomic localization of each family of MGE. Start and end positions refer to the corresponding contig. ICEs stand for Integrative Conjugative Elements, and IMEs stand for Integrative Mobilizable Elements.

Supplementary Data 11.
Description: a) Number of defense islands (DIs) per MAG per environment. Defense islands were defined as arrays of defense genes separated from one another by 10 genes or less and with a minimum of 5 genes pertaining to at least 3 different defense families. b) Number of defense islands (DIs) per MAG per environment. Defense islands were defined as arrays of defense systems separated from one another by 10 genes or less and with a minimum of 5 genes pertaining to at least 3 different defense families.

Supplementary Data 12.

Description: Values of odd ratios (OR) and corresponding two-sided Fisher's exact test P values for the all-against-all co-localizations of defense families inside defense islands.

Supplementary Data 13.
Description: Values of odd ratios (OR) and corresponding two-sided Fisher's exact test P-values for the all-against-all co-localizations of defense families inside defense islands. Data was split according to ecological (soil, marine) and geographical (human gut) contexts.

Supplementary Data 14.
Description: Quantification and classification of SNP and indel variants in soil and marine environments. Human data is not shown as we did not obtain globally significant O/E mutation values following chi-square test. Classification of SNPs as synonymous and non-synonymous is also shown. Ambiguous mutations refer to nonsynonymous and synonymous polymorphisms present at the site.

Supplementary Data 15.
Description: a) Variation in global dN/dS given by the Nei-Gojobori (NG) and Yang-Nielson (NY) methods for a selection of defense genes shown to harbor a significantly higher frequency of SNPs + Indels. P values correspond to a two-sided Fisher's exact test. b) Across gene profiles of dN/dS given by the Maximum Likelihood (Akaike Information Criterium), Nei-Gojobori (NG), and Yang-Nielson (NY) methods for a selection of three defense genes simultaneously present in soil and marine environments. P values correspond to a two-sided Fisher's exact test.
